# The Metabolic Network of Chilled Yak Meat During Storage Was Constructed Based on Metabolomics Technology

**DOI:** 10.3390/foods14183173

**Published:** 2025-09-11

**Authors:** Xingdong Wang, Shaoke Guo, Lin Xiong, Xiaoyun Wu, Pengjia Bao, Yandong Kang, Mengli Cao, Ziqiang Ding, Liyan Hu, Chunnian Liang, Jie Pei, Xian Guo

**Affiliations:** 1 Key Laboratory of Yak Breeding of Gansu Province, Lanzhou Institute of Husbandry and Pharmaceutical Sciences, Chinese Academy of Agricultural Sciences, Lanzhou 730050, China; wxd17339929758@163.com (X.W.); gsk1125@163.com (S.G.); xionglin@caas.cn (L.X.); wuxiaoyun@caas.cn (X.W.); baopengjia@caas.cn (P.B.); kangyandong0901@163.com (Y.K.); caomengliaaa@163.com (M.C.); dingziqiang1997@163.com (Z.D.); huliyan2020@163.com (L.H.); liangchuannian@caas.cn (C.L.); 2 Key Laboratory of Animal Genetics and Breeding on Tibetan Plateau, Ministry of Agriculture and Rural Affairs, Lanzhou 730050, China; 3 College of Life Science and Technology, Inner Mongolia Normal University, Hohhot 010022, China; 4 Key Laboratory of Biodiversity Conservation and Sustainable Utilization in Mongolian Plateau for College and University of Inner Mongolia Autonomous Region, Hohhot 010022, China

**Keywords:** GC-MS, HS-GC-IMS, flavor, proteins, nucleotide

## Abstract

Chilled yak meat is becoming more and more popular with the improvement in living standards, and the flavor of chilled meat is closely related to storage time. The effect of storage time on the flavor of chilled yak meat was explored in this study. We used GC-MS, HS-GC-IMS, and LC-MS/MS to detect changes in the metabolites in yak meat during storage at 4 °C and constructed storage time-dependent metabolite fingerprints of the yak meat. The results showed that low-temperature storage promoted the degradation of proteins and lipids, nucleotide release, and the production of the volatile compounds heptanal, octanal, n-nonanal, benzaldehyde, 2,3-pentanedione, 3-hydroxy-2-butanone, and 2-butanone. With an increase in the chilled storage time of yak meat, the total volatile basic nitrogen and total viable count of the meat were significantly increased. The short-term storage time of yak meat at 4 °C should not exceed 5 days.

## 1. Introduction

The yak (*Bos grunniens*) is a bovine species inhabiting regions at altitudes between 2500 m and 6000 m [1]. Therefore, it has evolved the anatomical and physiological characteristics of adaptation to extreme environments (high-altitude, low-temperature, high-ultraviolet-exposure, and low-oxygen), including a compact body, nonfunctional sweat glands, and a relatively small ratio of skin surface area to body weight [2]. Currently, there are approximately 17.5 million yaks worldwide, 94.4% of which are feed in China [3]. Yaks not only provide herders with necessities such as milk, meat, fur, and fuel [4] but also serve as a means of transportation. Thus, they are called “omnipotent livestock” [5]. Due to its sensory and nutritional characteristics, yak meat is widely consumed as a beef alternative in Hong Kong, Macao, and Western Europe [6]. Yak meat possesses higher protein content compared with the meat from other livestock and poultry [7], and it is also rich in amino acids, fatty acids, and minerals.

Fresh yak meat can easily undergo microbial contamination and lipid oxidation [8]. Frozen meat has prolonged shelf life, but frozen storage increases meat supply costs. Moreover, long-term frozen storage may cause significant changes in meat quality due to protein denaturation and fat oxidation, and frozen meat inevitably loses some juices after being thawed, resulting in a deterioration of its color and taste. Chilled meat refers to meat stored at 0–4 °C within 24 h post-slaughter. That temperature is maintained during the subsequent processing, transportation, and sale of the meat. Compared with fresh meat and frozen meat, chilled meat is favored by consumers because it possesses a delicate and delicious taste and abundant nutrients. Therefore, chilled meat consumption is expected to be the future of meat consumption [8]. However, chilled meat is easily affected by environmental conditions, microorganisms, and enzymes. These three factors may induce a decrease in the freshness of chilled meat, the spoilage of the meat, or even the production of toxic and harmful substances in the meat. Thus, they may cause food safety hazards and significant losses to meat-producing enterprises. As a result, the detection of meat freshness is very important. The changes in the levels of volatile substances during meat storage are an important indicator of the change in meat freshness [9].

Food flavor, determined by volatile and non-volatile compounds, directly influences sensory characteristics and consumer acceptance [10]. Gas chromatography–mass spectrometry (GC-MS) can accurately identify complex volatile compounds through a mass spectrometry library, covering a wide range of molecular weights [11]. However, GC-MS usually requires enrichment steps such as solid-phase microextraction, which requires a long sample pretreatment process. Some low-boiling-point compounds may be lost during the injection process, which limits its effectiveness [12]. The headspace–gas chromatography–ion-mobility spectrometry (HS-GC-IMS) method, based on differences in ion migration rates, has unique recognition capabilities for low-molecular-weight compounds (such as sulfides, short-chain aldehydes, and ketones) and isomers [13]. HS-GC-IMS does not require complex preprocessing and analysis only takes 10–30 min, making it suitable for rapid screening [12]. Therefore, combining HS-GC-IMS and GC-MS is necessary to comprehensively analyze the volatile compounds in food samples. The combination of the two can significantly improve the detection coverage and establish a more comprehensive fingerprint of volatile substances in food. The combination of metabolomic analysis, GC-MS, and liquid chromatography–mass spectrometry (LC-MS) has been used to comprehensively analyze the primary and secondary metabolites in dry-cured ham [14]. Microbial growth can lead to meat deterioration during storage [15]; thus, microbiological and biochemical changes during storage serve as indicators of meat quality and freshness [16]. There have been many studies on the metabolite changes in popular meats (mainly pork and chicken) during chilled storage. However, there are few reports on the metabolite changes in yak meat during chilled storage. Currently, most meat flavor studies rely on a single technology, such as GC-MS or GC-IMS, which exhibit varying sensitivities to volatile compounds [12]. In order to investigate the changes in volatile flavor compounds and non-volatile metabolites of yak meat during storage at 4 °C, this study used a HS-GC-IMS, GC-MS, and LC-MS/MS multi-platform combination to establish a fingerprint map of time-dependent metabolites in yak meat storage and analyzed the temporal evolution of key metabolic pathways such as protein degradation and lipid oxidation. By integrating multiple omics data to screen metabolic markers during the storage process of yak meat, theoretical support is provided for the cold chain standardization of yak meat products.

## 2. Materials and Methods

### 2.1. Ethical Statement

All animal care and use protocols detailed herein were performed according to the guidelines established by the China Council on Animal Care and the Ministry of Agriculture and Rural Affairs of the People’s Republic of China. The protocols were approved by the Animal Care and Use Committee of the Lanzhou Institute of Husbandry and Pharmaceutical Sciences of the Chinese Academy of Agricultural Sciences (approval no. SYXK-2014-0002).

### 2.2. Sample Collection

The meat samples were collected from Tianzhu Tibetan Autonomous County, Wuwei City, Gansu Province, China (coordinates: 103°3′6″ E, 37°9′30″ N, altitude: 2976.51 m). Six 4-year-old male Tianzhu white yaks that were similar in weight (248 ± 3 kg) and health status were selected. The yaks fasted for 12 h then were slaughtered according to standard procedures of the National Standards of China (GB/T19477-2018) [17]. The longissimus lumborum (1000 g) was collected from the area between the third and fourth ribs of each yak. The fat and connective tissue in the muscles were removed, with each yak carcass evenly divided into 8 portions and vacuum-sealed in aseptic bags. All yak meat was stored at 4 °C, then subjected to testing and analysis on specific chilled storage days (the 0th (D0), 1st (D1), 2nd (D2), 3rd (D3), 4th (D4), 5th (D5), 6th (D6), or 7th (D7) day).

### 2.3. Determination of the Total Viable Count (TVC), Total Volatile Basic Nitrogen (TVB-N), and Metabolites of Yak Meat

The TVC of yak meat was determined according to the National Standards of China (GB 4789.2-2016) [18], and the total number of colonies in the yak meat stored at 4 °C for a specific period was evaluated against the criteria for meat hygiene detailed in the standard. In a sterile environment, three replicates (10 g each) were collected from each of the 8 yak meat samples with different chilled storage periods that were prepared in Section 2.2. Each replicate was placed in a conical flask, and 100 mL of water was added to the flask. Then, the mixture was homogenized, incubated for 30 min, and filtered. The supernatant was used for subsequent analysis. The TVB-N of yak meat was determined by the semimicro Kjeldahl method according to the National Standards of China (GB 5009.228-2016) [19]. GC-MS, LC-MS/MS, and HS-GC-IMS were used to detect non-target metabolites in yak meat. The detection methods and parameters were based on the reports of Kang [20]. The detection details of the methods are shown in the Supplementary Materials.

### 2.4. Weighted Gene Co-Expression Network Analysis (WGCNA)

The selected metabolite set was screened, and low-quality metabolites that caused unstable results were removed. A similarity matrix and an adjacency matrix were constructed on the basis of metabolite expression profiles. Then, the adjacency matrix was successively converted into a topological overlap matrix and a dissimilarity topological overlap matrix, and the dissimilarity topological overlap matrix was used to build a hierarchical clustering tree. Metabolites were divided into modules, and the correlation between each module and each group (a specific time point of the 4 °C storage of yak meat) was evaluated. Core modules were identified using the WGCNA [21] package (version 1.69) in R software, and Kyoto Encyclopedia of Genes and Genomes (KEGG) enrichment analysis was performed on them.

### 2.5. Data Analysis

Principal component analysis (PCA), an unsupervised learning algorithm, and partial least squares discriminant analysis (PLS-DA), a supervised learning algorithm, were performed using SIMCA-P software (version 14.1; Umetrics, Sweden) to visualize the separation of yak meat samples with different chilled storage periods. An analysis of variance (ANOVA) was conducted using SPSS software (version 26.0). Metabolites with VIP (variable importance in projection) values > 1.5 (determined by PLS-DA) and *p*-values < 0.05 (determined by ANOVA) were considered as differential metabolites.

## 3. Results and Discussion

### 3.1. Changes in the TVB-N and TVC of Yak Meat During 4 °C Storage

**Figure 1:**
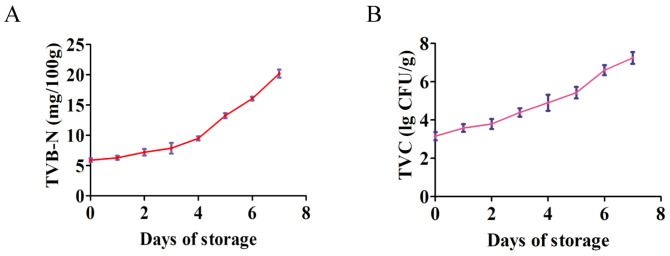
Changes in the main quality indexes of yak meat during 4 °C storage. The X-axis represents storage days, and the Y-axis indicates the measured values of TVB-N/TVC. (**A**) Changes in the TVB-N of yak meat during 4 °C storage. (**B**) Changes in the TVC of yak meat during 4 °C storage. Note: TVB-N (total volatile basic nitrogen indicates the TVB-N content (mg per 100 g of meat)); TVC (total viable count); n = 6.

Meat quality determines consumer acceptability and purchase desire [22]. TVB-N is an objective marker for the loss in the freshness and safety of meat-based products [23,24]. Figure 1A showed that there were no significant differences in TVB-N among D0, D1, D2, and D3 yak meat. However, the TVB-N of chilled yak meat gradually increased when the storage period of the meat was longer than 3 days. The proteolytic effect caused by spoilage bacteria and endogenous enzymes can lead to an increase in TVB-N [22]. The TVB-N values of D5, D6, and D7 yak meat were 13.3, 15.2, and 20.2 mg/100 g, respectively. Beef with TVB-N above 15 mg/100 g is deemed spoiled [25]. On the fourth day of storage, the TVB-N of the chicken stored at 4 °C is <15 mg/100 g (14.7 mg/100 g) [26]; on the sixth day of storage, the TVB-N of the chilled chicken is >15 mg/100 g. Due to the lack of test data for the fifth day of storage, Wen et al. [26] suggested that the storage period of chilled chicken should not exceed 4 days.

The microbial contamination of meat may occur during slaughtering and meat processing [27]. When meat is stored under certain conditions, the number of viable bacteria in it increases almost linearly. Therefore, the change in the number of viable bacteria can be used to determine the degree of meat contamination [28]. Figure 1B shows that the TVC of D0 yak meat is 103 CFU/g. With the extension of chilled storage time, the TVC of chilled yak meat gradually increased. The TVC of D5 yak meat was 105 CFU/g, while that of D6 yak meat was above 106 CFU/g. The TVC of fresh beef is below 4 lg CFU/g, the TVC of qualified beef is 4–6 lg CFU/g, and the TVC of deteriorated beef is above 6 lg CFU/g [29]. Therefore, D1 yak meat to D5 yak meat could be regarded as qualified meat, while D6 yak meat and D7 yak meat could be considered as deteriorated meat. Previous studies [26,28,30] and this study suggest that the storage time of the yak meat chilled at 4 °C should not exceed 5 days.

### 3.2. LC-MS/MS Analysis of the Non-Volatile Metabolites in the Yak Meat Stored at 4 °C

#### 3.2.1. Storage Time-Dependent Non-Volatile Metabolite Profiles of the Yak Meat Stored at 4 °C

There is a complex relationship between the flavor and volatile flavor compound composition of yak meat. As the precursor of volatile flavor compounds, metabolites have a significant influence on the flavor of yak meat [10]. The LC-MS/MS analysis of yak meat samples stored at 4 °C for different periods (D0 yak meat to D7 yak meat) showed that 5991 and 7070 fragment ions were obtained in the positive-ion mode (POS) and negative-ion mode (NEG), respectively. POS and NEG data were combined, and the retention time, accurate molecular weight, and MS/MS fragments of each compound in the combined dataset were compared with those of compounds in publicly available MS/MS databases (such as HMDB (Human Metabolome Database) and KEGG) and a self-built MS/MS database (BiotreeDB).

**Figure 2:**
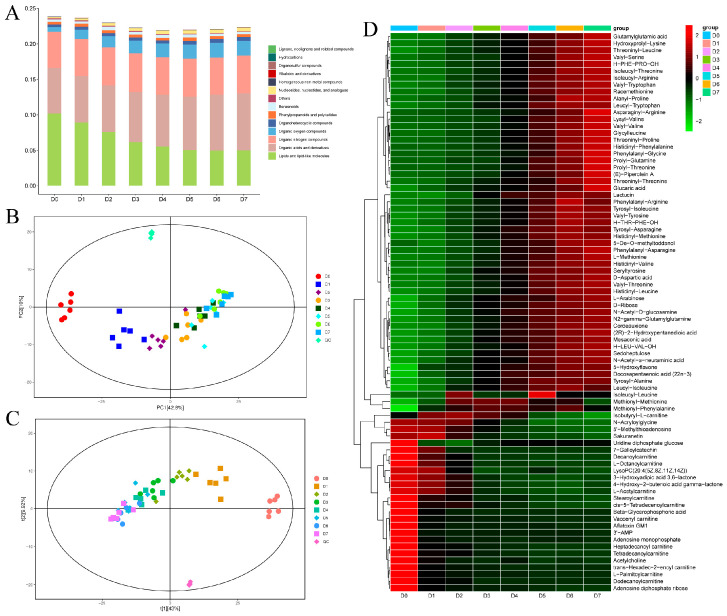
LC-MS/MS analysis of the non-volatile metabolites in yak meat samples stored at 4 °C for different periods. (**A**) Changes in the levels of different categories of metabolites during the 4 °C storage of yak meat. (**B**) A PCA score plot showing the separation of the yak meat samples. (**C**) A PLS-DA score plot showing the separation of the yak meat samples. (**D**) A heat map showing the metabolite differences among the yak meat samples. Note: Days of refrigerated meat storage: D0 (fresh) to D7 (7-day storage); n = 6.

A total of 674 non-volatile metabolites were detected in the yak meat samples (Supplementary Table S1). With the extension of the chilled storage time of yak meat, the level of lipids and lipid-like molecules gradually decreased, while the levels of organic acids and their derivatives increased (Figure 2A). Lipid oxidation occurs easily during yak meat storage and plays a key role in the formation of aroma during meat processing and storage [31]. Moderate lipid oxidation can produce an ideal meat flavor. For example, the characteristic flavor compounds (hexanal, 3-methyl-butanal, nonanal, and octanal) of the dry-cured ham from Dahe black pigs are formed through fatty acid oxidation and amino acid degradation [32]. Organic acids, which can be produced by fat oxidation, have unique umami and sour tastes [33]. The organic oxygen compounds and nucleosides, nucleotides, and their analogs in chilled yak meat gradually increased, which is consistent with the results of Fu et al.’s [8] study on the ice temperature preservation of yak meat. Nucleotides, which contribute to the umami taste of fish sauce, are taste-active substances present in most foods [34].

#### 3.2.2. PCA and PLS-DA

PCA and PLS-DA were used to explore the differences in metabolites in yak meat samples stored at 4 °C at different periods (D0 yak meat to D7 yak meat). The PCA score plot (Figure 2B) shows that the contributions of the first principal component (PC1) and second principal component (PC2) were 42.8% and 10%, respectively. Yak meat samples with the 4 °C storage period of 0–2 days (D0 yak meat to D2 yak meat) were clearly separated from yak meat samples with the 4 °C storage period of 3–7 days (D3 yak meat to D7 yak meat). However, yak meat samples with the 4 °C storage period of 3–7 days could not be clearly separated from each other, but they were also clustered regularly with the storage time. In order to understand the metabolite difference between groups, we constructed a PLS-DA score plot (Figure 2C). Each point in the PLS-DA score plot represents a sample; all points in the plot are within the 95% Hotelling’s T-squared confidence ellipse, indicating that there are no outliers in the samples and there is a clear separation between groups. Therefore, the flavor compounds in yak meat during chilled storage changed significantly.

#### 3.2.3. Changes in the Levels of Non-Volatile Metabolites in Chilled Yak Meat

A total of 81 differential metabolites were identified in yak meat samples stored at 4 °C for D0 yak meat to D7 yak meat. These differential metabolites included free amino acids and their derivatives, fatty acids and their derivatives, sugars and their derivatives, and secondary metabolites (such as alkaloids). Amino acids and their metabolites accounted for the largest proportion of differential abundance metabolites; this is consistent with the results of Fu et al.’s [8] study on the ice temperature preservation of yak meat. Fresh yak meat is rich in protein, and protein is easily degraded into small molecular peptide chains and amino acids by microorganisms during storage [8]. Therefore, the content of amino acids increases with the increase in storage time. Some of these amino acids are important flavor substances in meat, giving a sweet, sour, salty, bitter, umami, or kokumi taste [34,35]. Aspartate, alanine, glycine, and glutamic acid are amino acids with an umami taste [36]. Leucine and isoleucine can react with dicarbonyl compounds formed in Maillard reactions to produce 2- and 3-methylbutyraldehyde meat odorants, thus affecting the flavor of beef [8].

A heat map was constructed on the basis of the results of hierarchical cluster analysis (Figure 2D), and it shows there were 13 fatty acids in all 81 differential metabolites. The levels of docosapentaenoic acid (22n−3) and mesaconic acid were increased significantly on the sixth and seventh days of the 4 °C storage of yak meat (D6 and D7, respectively), but the levels of the other 11 fatty acids were higher at the early stage of the chilled storage, especially the 0th day of the chilled storage (D0). Fatty acids are the precursor of the characteristic flavor compounds of pork [37]. The synthesis and degradation of fatty acids have a significant impact on downstream volatile flavor compounds [38]. For example, the degradation of fatty acids leads to the formation of odorants in chicken soup and meat [39]. Short-chain fatty acids directly affect the flavor of ham, and long-chain fatty acids can be converted into various flavor ingredients, like alcohols and esters, by oxidation reactions [40]. Due to the conversion of fatty acids into flavor compounds, the flavor of yak meat was gradually enriched.

There were three nucleotides, two organic acids, and two organic oxygen compounds in the eight-one differential metabolites. The level of nucleotides in the early storage period was higher than that in the later storage period, while the levels of organic acids and organic oxygen compounds on D6 and D7 were higher than those on D0 to D5. During meat spoilage, the changes in organic acids and their derivatives have relatively minor effects on meat quality. Organic acids lead to a decrease in pH and contribute to a sour or soapy flavor [8].

### 3.3. HS-GC-IMS Analysis of the VOCs in the Yak Meat Stored at 4 °C

#### 3.3.1. Storage Time-Dependent Topographic Plots of Chilled Yak Meat

**Figure 3:**
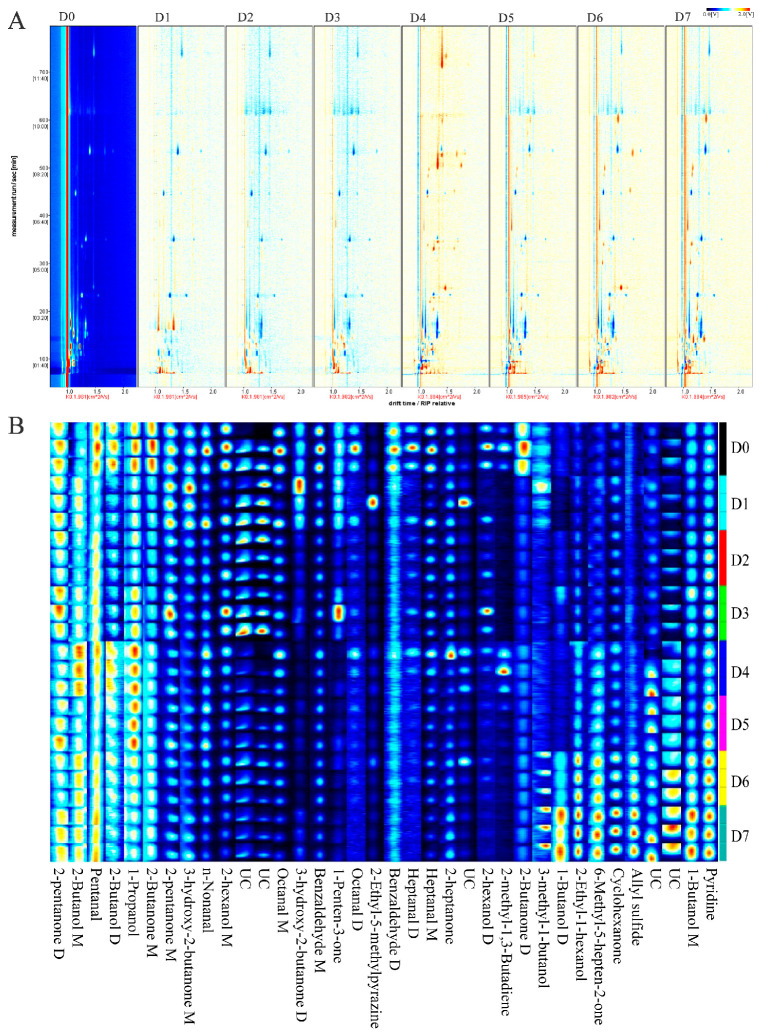
HS-GC-IMS analysis of the VOCs in yak meat samples stored at 4 °C for different periods. (**A**) Two-dimensional topographic plots of the yak meat samples. D0 yak meat was used as the background plot. The X-axis represents the storage days, and the Y-axis indicates the GC-IMS ion difference in volatile flavor substances. (**B**) Fingerprint information of the characteristic flavor of yak meat samples generated by a Gallery plot from different periods. M: monomer; D: dimer. Note: days of refrigerated meat storage: D0 (fresh) to D7 (7-day storage).

Difference plots (Figure 3A) were obtained by topographic plot derivation (the topographic plot of D0 yak meat was used as the background plot), and the changes in VOCs during the 4 °C storage of yak meat were evaluated. Red signals appeared in the differential plots of D1, D2, and D3 yak meat in the retention time range of 50–250 s and the drift time range of 1.0–1.3. On the other hand, red signals appeared in the differential plots of D4, D5, D6, and D7 yak meat in the retention time range of 50–600 s and the drift time range of 1.0–1.5 s. The intensity of the blue signals appearing in the retention time range of 50–250 s increased with an increase in the chilled storage time of yak meat. These results indicate that a large number of VOCs are formed during the chilled storage of yak meat.

#### 3.3.2. VOC Variations in Chilled Yak Meat

A total of 36 VOCs were identified by HS-GC-IMS in yak meat stored at 4 °C for different periods (D0 yak meat to D7 yak meat). These VOCs included nine alcohols, eight aldehydes, ten ketones, one ether, one pyridine, one pyrazine, one allyl sulfide, and five unknown compounds (Figure 3B and Supplementary Table S2). Most aldehydes are derived from the oxidative hydrolysis of fats, and a very small portion of them are derived from the Meladic reaction of sugars [41]. High levels of aldehydes were found in D0 yak meat. With the increase in the chilled storage time of yak meat, the levels of aldehydes gradually decreased except for the benzaldehyde dimer. Aldehydes which are the products of the oxidative degradation of fats have a low odor threshold and can produce a wide range of fragrances [41]. Benzaldehyde has almond-like, fruity, aromatic, and sweet aromas [12], and its level gradually decreased with an increase in the chilled storage time of yak meat. The levels of octanal, heptanal, and n-nonanal gradually decreased too. Heptanal and octanal with a low odor threshold can produce pleasant meaty, grassy, and fruity aromas [42]. Nonanal, with a low odor threshold, can endow citrus-like and green aromas [43,44].

The levels of 2-ethyl-1-hexanol and 1-butanol increased gradually with an increase in the chilled storage time of yak meat. The levels of 2-butanol dimer and 1-butanol dimer remained unchanged on D0 to D5 and increased rapidly on D6 and D7. The level of 3-methyl-1-butanol decreased gradually with an increase in the chilled storage time of yak meat. The level of 2-butanol dimer on D0 was higher than those of the VOC on D1 to D7, and the level of the VOC remained unchanged on D1 to D7. The levels of 2-hexanol, 2-hexanol dimer, and 1-propanol decreased, increased, and then decreased with an increase in the chilled storage time of yak meat. Alcohols are usually derived from the oxidative decomposition of fats [45]. High concentrations of alcohols produce herb-like, woody, and fatty aromas in meat products [42]. Hexanol is the main VOC in unfermented black beans, contributing to beany and fruity flavors [12]. The levels of ethanol and 2-octanol gradually increased with an increase in the chilled storage time of yak meat. Octanol is formed by the oxidation of myristic acid, linoleic acid, palmitoleic acid, or oleic acid [42]. It contributes to the green and woody aromas of ham [46]. Compared with aldehydes, alcohols have a higher odor threshold and a smaller contribution to the flavor of meat products [31].

Ketones are generally considered to be the secondary products formed during lipid oxidation, alkane degradation, and secondary alcohol dehydrogenation [15]. They are usually associated with creamy and fruity flavors, but they also contribute significantly to the aroma of meat, although their flavor threshold is slightly higher than that of aldehydes [31,41]. The levels of cyclohexanone and 6-methyl-5-hepten-2-one were increased with an increase in the chilled storage time of yak meat. The level of cyclohexanone remained unchanged on D0 to D5 but increased rapidly on D6 and D7. The levels of 2-butanone and 2-butanone dimer on D0 were higher than those of the VOCs on D1 to D7 and remained unchanged on D1 to D7. With an increase in the chilled storage time of yak meat, the levels of 2-pentanone, 1-penten-3-one, and 3-hydroxy-2-butanone gradually decreased, while the levels of the other ketones remained unchanged. 3-hydroxy-2-butanone, also known as acetoin, has a pleasant milky flavor and results from the Maillard reaction, possibly from oleic acid oxidation or glucose decomposition [41].

The level of 2-ethyl-5-methylpyrazine gradually decreased with an increase in the chilled storage time of yak meat. The aroma of pyrazine is similar to that of cooked/roasted beef [47]. While the level of pyridine gradually increased. The level of allyl sulfide remained unchanged on D0 to D5, but it increased significantly on D6 and D7.

### 3.4. GC-MS Analysis of the VOCs in Chilled Yak Meat

**Figure 4:**
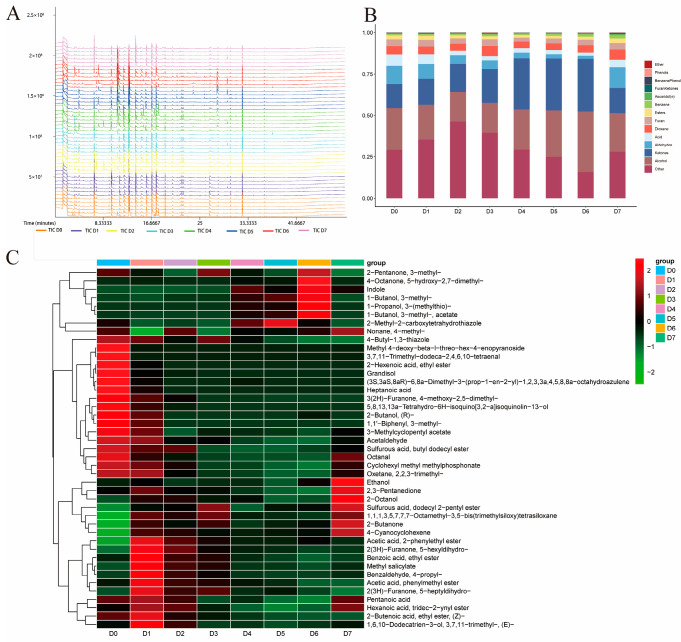
GC-MS analysis of the VOCs in yak meat samples stored at 4 °C for different periods. (**A**) Total ion chromatograms of the yak meat samples. (**B**) Changes in the levels of different categories of VOCs during the 4 °C storage of yak meat. (**C**) A heat map showing the metabolite differences among the yak meat samples. Note: days of refrigerated meat storage: D0 (fresh) to D7 (7-day storage).

GC-MS analysis showed that the number and area of VOC peaks increased with an increase in the chilled storage time of yak meat (Figure 4A), indicating that chilled storage leads to the formation of VOCs in yak meat. A total of 388 VOCs were identified by GC-MS (Supplementary Table S3). With an increase in the chilled storage time of yak meat, the levels of benzene, ascaridole, and ketones gradually increased, but the level of ketones decreased sharply on D7 (Figure 4B). The levels of acids and aldehydes gradually decreased on D0 to D6, but they increased sharply on D7. Aldehydes are important indicators of meat spoilage, spoilage, and flavor changes [8]. In the process of beef spoilage, the amination and transamination of aldehydes and the decarboxylation of free amino acids will produce amines, which will lead to the spoilage of beef [8].

The level of alcohols decreased on D0 to D2, increased gradually on D3 to D6, and decreased sharply on D7. Among the 388 metabolites, 43 metabolites were identified as differential metabolites (Figure 4C). These differential metabolites included 12 esters, 7 alcohols, 7 ketones, 4 aldehydes, 2 acids, 3 alkanes, and 8 others. The two acids were mainly detected in the early chilled storage stage of yak meat. The level of 2-butanone increased significantly on D7. Studies have shown that 2-butanone can be used as a spoilage indicator [24], suggesting the spoilage of chilled yak meat on D7. The level of 2-heptanone increased on D0 to D4 and then decreased on D5 to D7. As the degradation product of linoleic acid, 2-heptanone contributes to blue cheese, creamy, or fruity flavors [42,48]. The level of 3-methylbutyl acetate, which is an ester with a banana-like fragrance [12], increased significantly on D5 and remained high on D6 and D7. Esters from short-chain fatty acids have a low flavor threshold and sweet and fruity flavors, esters from long-chain fatty acids have a nutty flavor, while aromatic esters have a high flavor threshold and a slight contribution to flavor [41]. GC-MS and GC-IMS have different degrees of sensitivity to volatile substances [12]. This could be because the number of detected peaks in GC-IMS can vary widely depending on factors such as the type of sample, the chromatographic conditions, and the sensitivity of the instruments [41]. Therefore, there were some differences between the results obtained by GS-MS and those obtained by GC-IMS.

### 3.5. WGCNA

**Figure 5:**
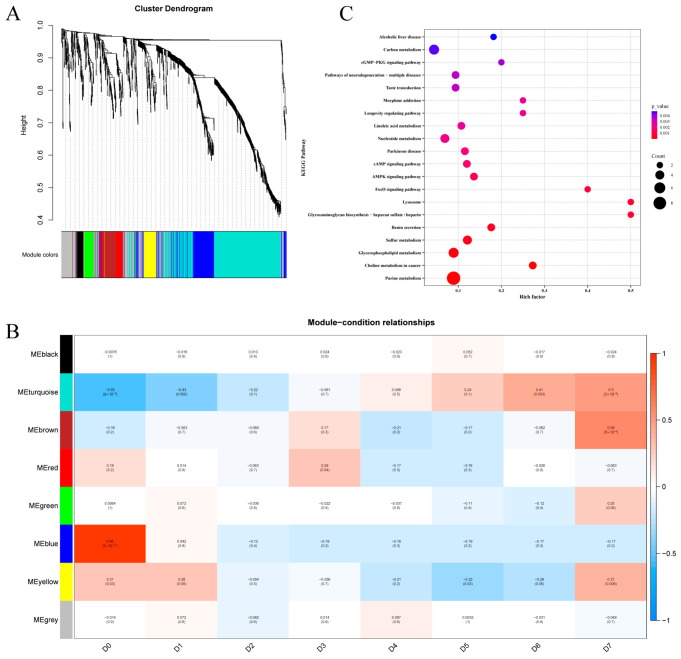
WGCNA of the metabolites detected by HS-GC-IMS, GC-MS, and LC-MS in yak meat samples stored at 4 °C for different periods. (**A**) A hierarchical clustering tree showing co-expression modules. (**B**) Correlations between the co-expression modules and specific time points of the 4 °C storage of yak meat. Red represents a positive correlation and blue represents a negative correlation. (**C**) KEGG enrichment analysis of the metabolites in blue and yellow modules. Note: days of refrigerated meat storage: D0 (fresh) to D7 (7-day storage).

WGCNA was conducted on HS-GC-IMS, GC-MS, and LC-MS/MS data from 48 yak meat samples with eight different periods of 4 °C storage (six samples for each of D0 yak meat to D7 yak meat). After modules with similarity greater than 0.75 were merged, eight co-expression modules were obtained (Figure 5A). In order to find core modules (modules with biological significance), the correlation between each co-expression module and each time point of the 4 °C storage of yak meat was evaluated (Figure 5B). The blue module was highly correlated with D0 (r = 0.96, *p* = 5 × 10^−27^). The turquoise module was closely correlated with D6 (r = 0.41, *p* = 0.003) and D7 (r = 0.50, *p* = 3 ×10^−4^). The red module was highly correlated with D3 (r = 0.29, *p* = 0.04). The yellow module was highly correlated with D0 (r = 0.31, *p* = 0.03) and D1 (r = 0.28, *p* = 0.05). The brown module was highly correlated with D7 (r = 0.59, *p* = 9 × 10^–6^). The five modules were used as storage time-specific modules in the analysis of the biological functions of core modules.

### 3.6. KEGG Enrichment Analysis of Storage Time-Specific Modules

**Figure 6:**
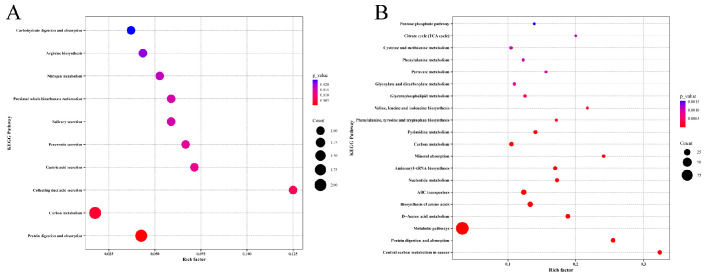
KEGG enrichment analysis of the metabolites in different modules. KEGG enrichment analysis of (**A**) the metabolites in the red module and (**B**) the metabolites in the brown and turquoise modules.

To further understand the pathways associated with the differentially abundant metabolites, the five storage time-specific modules identified in Section 3.5 were selected for KEGG enrichment analysis. Signaling pathways including purine metabolism, sulfur metabolism, and glycerophospholipid metabolism were significantly correlated with D0 and D1 (Figure 5C). Protein degradation in yak meat mainly occurs through purine metabolism [8]. This is consistent with the results of the LC-MS/MS analysis of the non-volatile metabolites in chilled yak meat (Section 3.2.3). Phospholipids can be hydrolyzed by phospholipases into glycerol and fatty acids. Signaling pathways including carbon metabolism and protein digestion and absorption were significantly associated with D3 (Figure 6A). Metabolic pathways were significantly associated with D6 and D7 (Figure 6B). This supported the LC-MS/MS results that peptides and amino acids were mainly concentrated in the later stage of the chilled storage of yak meat.

These KEGG enrichment analysis results reveal the molecular mechanisms underlying the dynamic changes in the metabolic pathways of yak meat during different storage stages. The significant enrichment of the purine metabolism pathway in the D0—D1 stage confirms the active state of the protein hydrolase system in the early storage stage, which is consistent with the characteristics of ATP metabolite accumulation (such as inosine acid) in post-mortem muscles [49]. It is worth noting that the synchronous activation of the sulfur metabolism pathway may reflect the degradation process of sulfur-containing amino acids (such as cysteine and methionine), which are both flavor precursors and closely related to oxidative stress regulation [50]. The activation of glycerophospholipid metabolism reflects the degradation process of cell membrane phospholipids, and the free fatty acids produced can serve as precursors for flavor compounds or trigger lipid oxidation chain reactions. In addition, the association between this pathway and signaling pathways such as MAPK suggests the possibility of a synergistic regulation mechanism between metabolism and signal transduction, which in turn affects the apoptosis process of muscle cells [51]. The activation of carbon metabolism and protein digestion and absorption pathways in the D3 stage is consistent with the accumulation of lactate produced by anaerobic glycolysis of muscle glycogen. This metabolic transition leads to a decrease in pH and affects the water-holding capacity of meat. The enrichment of branched-chain amino acid and aromatic amino acid metabolic pathways in the later stage (D6-D7) confirms the degradation of myofibrillar proteins mediated by the calpain system [52] and also suggests that microorganisms or endogenous enzyme systems in the later stage of refrigeration may further affect meat flavor through branched-chain amino acid metabolic pathways such as valine/leucine. In addition, the time-specific distribution of these pathways provides a framework for understanding key metabolic events at different refrigeration stages: in the early stages (D0-D1), energy-related metabolites (such as purines) and membrane stability (such as glycerophospholipids) are predominant, while in the later stages (D6-D7), there is a shift towards the generation of protein breakdown end products.

## 4. Conclusions

With an increase in the chilled storage (4 °C storage) time of yak meat, the TVB-N and TVC of the meat increased significantly. The levels of non-volatile substances such as lipids and lipid-like molecules gradually decreased, whereas the levels of volatile/flavor substances such as organic acids and their derivatives; organic oxygen compounds; amino acids and their derivatives; and nucleosides, nucleotides, and their analogs gradually increased. The levels of alcohols, ketones, and esters decreased gradually on D1 to D6 and increased sharply on D7, while the level of aldehydes gradually increased with an increase in the chilled storage time of yak meat. A high concentration of 2-butanone (which was selected as a spoilage marker) was detected on D6 and D7. This study showed that the short-term storage time of yak meat at 4 °C should not exceed 5 days. However, due to the lack of detailed data on microorganisms in meat, it is impossible to determine which microbial activities are the results of yak meat spoilage. It can only be judged by metabolite levels. In the later stage, the types of microorganisms during yak meat storage can be detected to monitor the deterioration process of meat.

## Data Availability

The original contributions presented in this study are included in this article/Supplementary Materials. Further inquiries can be directed to the corresponding authors.

## Supplementary Materials

The following supporting information can be downloaded at: https://www.mdpi.com/article/10.3390/foods14183173/s1, Table S1: Detection results of non-volatile compounds; Table S2: Detection results of HS-GC-IMS; Table S3. Detection results of GC-TOF-MS.
